# A method to promote safe cycling powered by large language models and AI agents

**DOI:** 10.1016/j.mex.2024.102880

**Published:** 2024-07-26

**Authors:** Daniel G. Costa, Ivanovitch Silva, Morsinaldo Medeiros, João Carlos N. Bittencourt, Matheus Andrade

**Affiliations:** a SYSTEC, University of Porto, Porto, Portugal; b Federal University of Rio Grande do Norte, Natal, Brazil; c CETEC, Federal University of Recôncavo da Bahia, Cruz das Almas, Brazil; d PDEEC, University of Porto, Porto, Portugal

**Keywords:** Artificial Intelligence, Open data, Urban mobility, Smart cities, SafeCycle-Assist

## Abstract

This paper presents a novel information generation methodology to support safer cycling patterns in urban environments, leveraging for that Large Language Models (LLMs), AI-based agents, and open geospatial data. By processing multiple files containing previously computed urban risk levels and existing mobility infrastructure, which are generated by exploiting open data sources, our method exploits multi-layer data preprocessing procedures and prompt engineering to create easy-to-use, user-friendly assistive systems that are able to provide useful information concerning cycling safety. Through a well-defined processing pipeline based on Data Ingestion and Preparation, Agents Orchestration, and Decision Execution methodological steps, our method shows how to integrate open-source tools and datasets, ensuring reproducibility and accessibility for urban planners and cyclists. Moreover, an AI agent is also provided, which fully implements our method and acts as a proof-of-concept implementation. This paper demonstrates the effectiveness of our method in enhancing cycling safety and urban mobility planning.

• A novel method that combines LLMs and AI agents is defined to enhance the processing of multi-domain open geospatial data, potentially promoting cycling safety.
• It integrates urban risk data and cycling infrastructure for a more comprehensive understanding of cycling resources, which become accessible by textual or audio prompts.

## Graphical abstract

**Figure:**
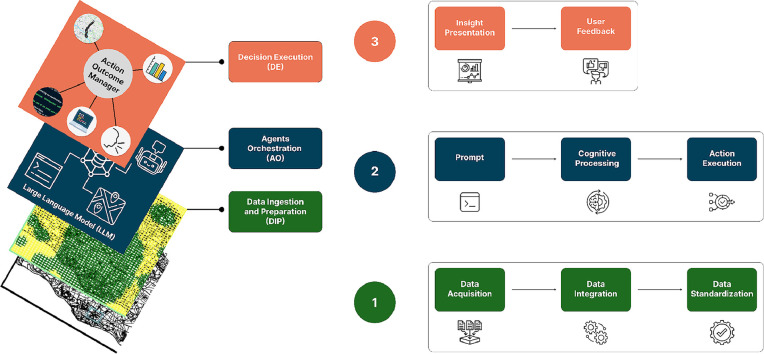

Specifications table

**Table:** 

Subject area:	Computer Science
More specific subject area:	*Artificial Intelligence, Geospatial data analyses*
Name of your method:	SafeCycle-Assist
Name and reference of original method:	Peixoto, J. P. J., Costa, D. G., da Franca Rocha, W. D. J., Portugal, P., and Vasques, F. (2023). CityZones: A geospatial multi-tier software tool to compute urban risk zones. SoftwareX, 23, 101409. https://doi.org/10.1016/j.softx.2023.101409 Medeiros, T., Medeiros, M., Azevedo, M., Silva, M., Silva, I., and Costa, D. G. (2023). Analysis of language-model-powered chatbots for query resolution in pdf-based automotive manuals. Vehicles, 5(4), 1384–1399. https://doi.org/10.3390/vehicles5040076
Resource availability:	https://github.com/conect2ai/METHODX2024-safecycle-assist http://cityzones.fe.up.pt

## Background

Cycling is an increasingly popular mode of transportation in urban areas due to its environmental benefits, health advantages, and potential to alleviate traffic congestion [1]. However, safety concerns remain a significant barrier to widespread adoption, mostly because cyclists are inherently more vulnerable to a myriad of accidents [1,2]. The perception of risk, influenced by factors such as traffic density, road conditions, crime rates, among others, should play a crucial role in some routines, such as route selection and parking, changing the usual reasoning centered only on existing cycling infrastructure (bike lanes, sharing points, etc.). Addressing such safety concerns through proper data gathering, processing, and actioning can significantly enhance the cycling experience and encourage more people to cycle [3].

After a cycling accident, some form of response service will be activated to attend to the victims, which may be manifested, for example, in the form of ambulance dispatching and police intervention to reorganize traffic. Such services are supported by emergency response infrastructures (PoI - Point of Interest), notably hospitals, fire brigades, and police stations, which will be fundamental to indicate the vulnerability of an area when an emergency happens. The CityZones tool was developed based on the available PoIs extracted from the OpenStreetMap platform [3], being one of the sources employed in our methodology. That tool divides a city into a number of square-shaped disjoint zones, each one classified in one risk level ranging from 1 (lowest risk) to 3 (highest risk), with the commutative distance of a zone to all PoIs being used to compute its urban risk. The works in [4,5] demonstrate such computation, which employs normalization and natural logarithm to perform risk assessment of any city.

Urban risk levels associated with mitigation actions after accidents are already valuable for many urban planning scenarios. However, the practical exploitation of this data may be challenging for citizens and cyclists alike. Therefore, implementing multi-layer methods to allow information retrieval may significantly amplify the practical benefits of such data [6]. In this context, recent advancements in artificial intelligence, particularly Large Language Models (LLMs) and AI agents, have opened new avenues for processing and interpreting complex datasets [7]. These models, which excel in understanding and generating human-like text, can be employed to analyze urban data and generate useful information.

This paper describes a novel methodology that employs LLM and AI agents to generate information on cycling safety in accordance with risk levels (derived from the CityZones tool) and cycling infrastructure (derived from OpenStreetMap). The core of our methodology, designated as SafeCycle-Assist, entails the processing of multiple files representing any given city selected by a user. Subsequently, based on the input files and a suitable pre-processing phase, users may create any prompt (e.g. textual or audio queries) and receive textual responses regarding risk levels on a path or specific coordinates.

The integration of data-driven urban risk perception with open-source tools and AI paradigms enables the development of a methodology that is effective, reproducible and accessible to a broad audience, thereby supporting safer and more sustainable smart cycling.

## Method details

**Fig. 1:**
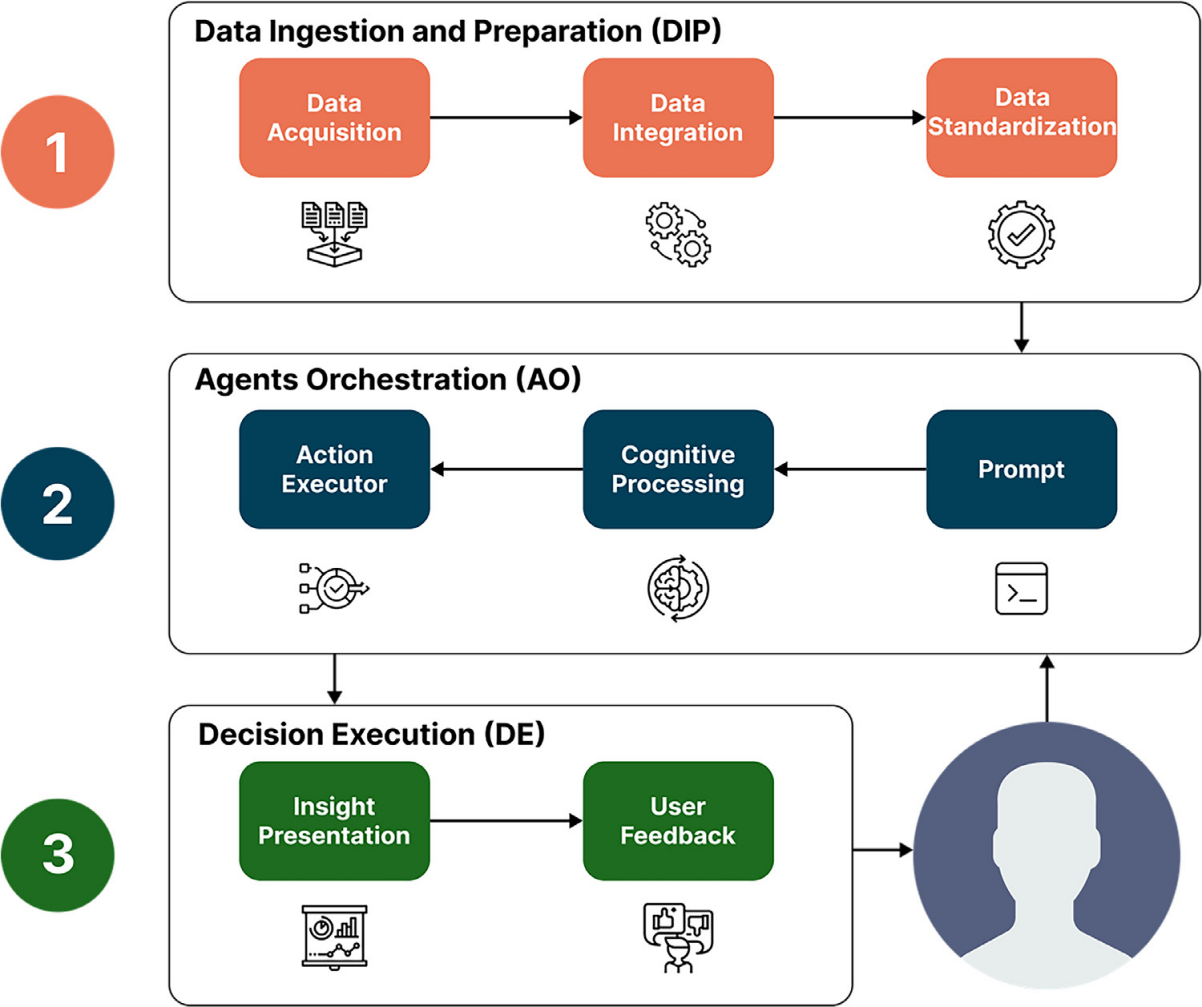
The processing guidelines and data flow of the SafeCycle-Assist method.

The SafeCycle-Assist method is divided into three major processing blocks, as depicted in Fig. 1: 1) Data Ingestion and Preparation (DIP), 2) Agents Orchestration (AO), and 3) Decision Execution (DE). These blocks are executed sequentially.

The summarization of the proposed methodological steps is described as follows:

1. Data Ingestion and Preparation (DIP): The first block begins with the **Data Acquisition** step, which is based on the collection of data generated by the CityZones tool and data exported from the OpenStreetMap. The CityZones exports data in the form of a CSV file, while the OpenStreetMap is accessed through the open-source OSMnx API [8]. The CSV file provides geospatial data of urban zones, which are modeled as small disjoint squares with a GPS coordinate representing their center point. Each zone is classified into one of three possible classes: 3 (high risk), 2 (medium risk), and 1 (low risk) [4,5]. In contrast, the data retrieved by the OSMnx API contain all cycle paths in the same city defined by the CityZones. Subsequently, these data are processed in the **Data Integration** step, resulting in a new geospatial understanding of the modeled urban area. Finally, the **Data Standardization** step is responsible for all required data normalization, the removal of unnecessary data, and the preparation of data for further processing in our methodology pipeline. This sanitization process is necessary, being quite common in similar processing tasks in this area [9].
2. Agents Orchestration (AO): The second block is responsible for the intended processing tasks. The **Prompt** step receives direct inputs from the user, who is typically a cyclist seeking useful cycling information. Such input is in the form of a textual query, but there is no requirement for the actual format. The **Cognitive Processing** step then performs prompt engineering, which is devoted to defining a context to guide the processing of our defined agent. In order to achieve this, an instruction file was defined, referred to as *context.md,* and released in the official method repository for reproducibility. At this stage, a Large Language Model (LLM) is adopted to interpret the defined context and the user input in order to take the appropriate actions. Finally, the **Action Executor** step takes place, acting as instructed by the previous step.
3. Decision Execution (DE): The last block of the process consists of two steps: data visualization and user feedback. The **Insight Presentation** step displays the generated output in a visible format (e.g., GeoJSON, audio, text, images, or any viable media). The **User Feedback** step interacts with users and provides additional suitable information.

To facilitate the practical adoption of our proposed method, we have developed a specialized agent-based tool that acts as a bridge between the CityZones/OpenStreetMap tools and the LLM model. This agent streamlines data importation, processing, and visualization, providing users with an intuitive interface to receive input parameters and produce useful outputs. Moreover, the methodology was applied to the city of Porto, Portugal, to generate useful information about safer cycling for the defined instruction configurations. The developed tool is ready to use and can be considered an additional guideline to further support the reproduction of our method.

## Method validation

Although our CiclySafe-Assist method is generic and easily adaptable for different contexts, an agent-based tool was developed to demonstrate its practical applicability in real scenarios and to support the creation of new tools based on our methodology. That tool was developed in accordance with the methodological steps depicted in Fig. 1 and is fully documented in the corresponding GitHub repository. Additionally, a Jupyter Notebook file (example.ipynb) has been added to the project repository. This notebook provides a detailed step-by-step example demonstrating the methodology. In order to facilitate the reproducibility and free availability of the tool, open-source libraries were adopted for both LLM [10] and AI agent [11] developments.

**Fig. 2:**
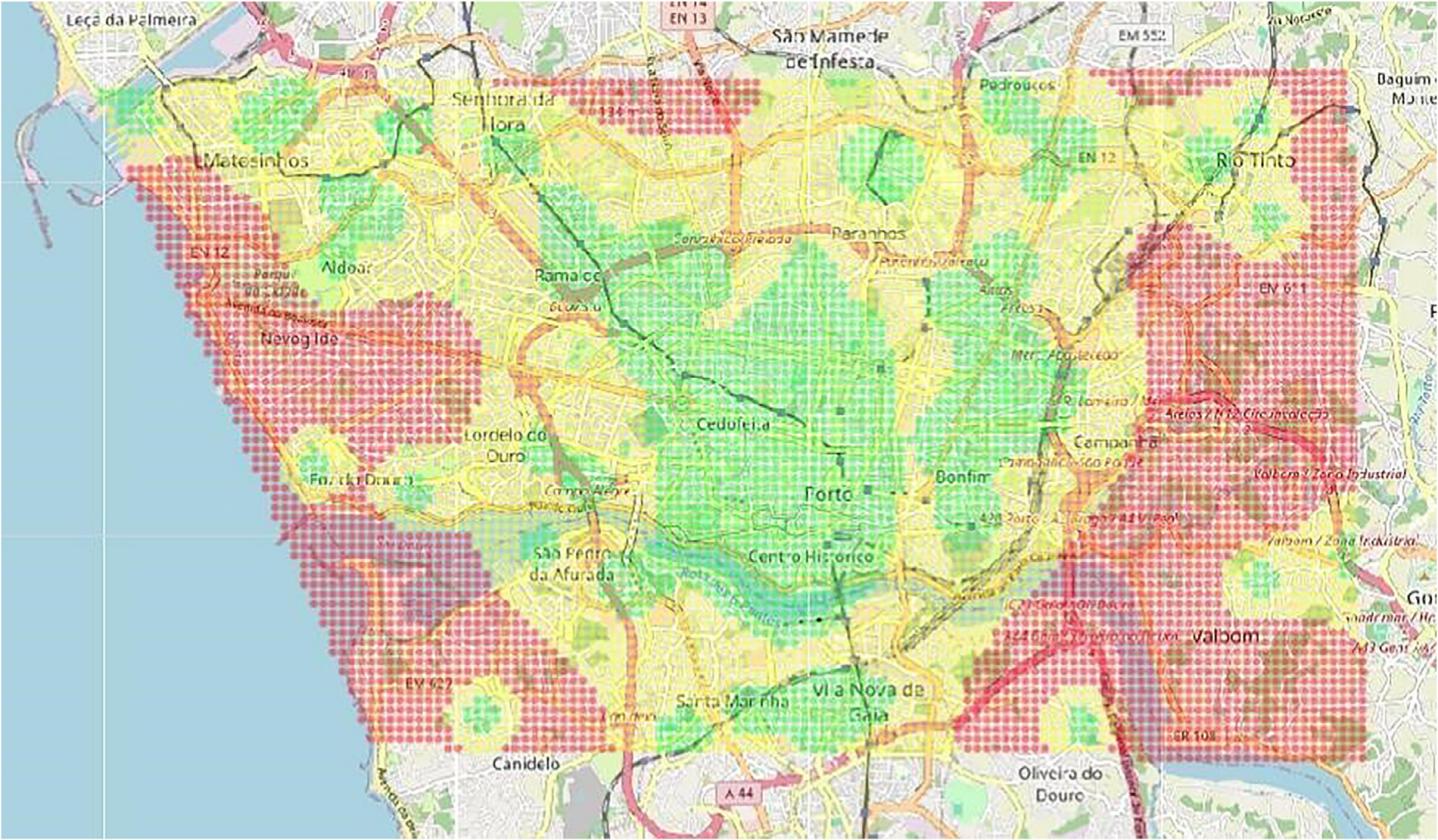
The considered risk computation for the city of Porto, Portugal, where red is the highest risk and green is the lowest. The GPS coordinates (latitude, longitude) of the defined bounding box are (41.11791317318376, −8.718653955363617), (41.19568943989527, −8.718653955363617), (41.11791317318376, −8.527985046673981), and (41.19568943989527, −8.527985046673981).

The developed tool considered the computed urban risk levels for the city of Porto, Portugal. However, the CiclySafe-Assist method is suitable for any urban area. As previously stated, the risk levels are exported from the CityZones tool, with a visual representation of the risk zones depicted in Fig. 2. This same urban area is used to retrieve cycling infrastructure data from the OpenStreetMap database.

The tool repository defines a reference contextual description that guides the assistive capacity of the data. Based on that and the input files from both CityZones and OpenStreetMap tools, we could demonstrate the practical impact of our method, particularly showcasing its ability to mitigate risks when promoting safer urban cycling practices. Overall, the extraction of useful information to enhance bikeability is an increasing practice in modern cities [12], facilitating the achievement of sustainable development goals.

The employed resources and significant configuration options of the developed tool are presented in Listing 1. This is a pseudocode describing the overall required steps for each of the components that comprise the SafeCycle-Assist method, as well as the expected input and output data.

**Listing 1:** A pseudocode representation of the system's primary function and the structure of the agent toolset employed during the experiments.

**Agent tools specification**	**Structure of the SafeCycle-assist agent**
**Structure of Risk Point Tool** Input Source Output Risk level Main Load file containing geolocations and associated risks For each geolocation in file Calculate the Haversine distance to the source location Identify the geolocation with the shortest distance Return risk associated with this geolocation **Structure of Risk Trip Tool** Input Source Destination Output Path risk levels Main Load file containing geolocations and associated risks Load city graph network Find nearest node to source Find nearest node to destination Compute the shortest path using Dijkstra's algorithm For each node in the shortest path Convert node to geolocation Find the nearest risk zone using Haversine distance Assign risk to the node Compute percentage of each risk type Return the percentages associated with each risk on route	Initialize Inputs OpenAI API Key GPT-model Model temperature Tools Risk of a point Risk of a trip Chat prompt template User prompt (with at least one coordinate) Outputs Targeted evaluation Process Configure GPT-model Define inputs GPT-model: gpt-3.5-turbo-0125 Temperature: 0 Define prompt conversation Message Role: agent Content: “You are an agent responsible for assisting users in computing cycling routes based on risk levels. Your task is to analyze the computed risk levels.” (context.md) Role: user Content: User prompt Initialize agent Bind tools Configure output parser Run agent executor Select a tool according to the prompt context Evaluate tool output with GPT-model Return Evaluation

The functions described in Listing 1 are available as a Python source code on the GitHub repository listed in the Supplementary Materials section. It is also worth noting that the actual agent content message was crafted with great care to reduce model hallucination by explicitly describing how the user prompts must be handled.

Following the implementation of our method, a series of tests were conducted to validate its applicability in extracting useful information about safe cycling. Listing 2 presents two practical examples of user inputs and the corresponding output.

**Listing 2:** Usage examples of the developed agent-based tool.

**User prompt input**	**Output**
⁠I am planning a cycling trip that will cross the point at latitude 41.1246 and longitude −8.5717. What is the risk associated with this zone?	The risk associated with the point at latitude 41.1246 and longitude −8.5717 is classified as high risk (Level 3). This indicates that the area poses significant hazards and is recommended only for very experienced cyclists. It is far from emergency services and other emergency-related amenities. Given the high-risk level, I recommend considering an alternative route for your cycling trip to ensure your safety. If you provide me with the starting and ending points of your trip, I can assist you in evaluating a safer route. Remember to wear appropriate safety gear, check the weather conditions, carry a basic repair kit, stay hydrated, and share your route details with someone in case of an emergency.
What is the risk of a cycling trip starting from a point with latitude 41.1246 and longitude −8.5717, ending at latitude 41.1570 and longitude −8.6393?	The computed risk levels for your cycling trip are as follows: • ⁠Low Risk: 53.79 % • ⁠Medium Risk: 30.30 % • ⁠High Risk: 15.91 % Based on these percentages, the trip is categorized as low risk with a significant margin over medium and high risks. It is considered safe for most cyclists, including beginners. The path is within close proximity to emergency services, ensuring a safer journey. Remember to wear appropriate safety gear, stay on designated cycling lanes, and enjoy your ride!
I am a tourist in Porto, Portugal, and I would like to travel using my bicycle. I am starting from a place I really enjoyed visiting, with coordinates latitude 41.1784 and longitude −8.5962. I would like to go to a spot recommended by a friend for photos, with coordinates latitude 41.1470 and longitude −8.6263. Could you help me with the risk associated of this journey?	The computed risk levels for your journey from the starting point at latitude 41.1784, longitude −8.5962 to the destination at latitude 41.1470, longitude −8.6263 are as follows: - Low Risk: 58.87 % - Medium Risk: 41.13 % - High Risk: 0.00 % Based on the analysis, the route is predominantly low risk with a small portion of medium risk. Here is a tailored response based on the risk levels: - Low Risk (1): “The route from your starting point to the recommended photo spot is considered low risk, making it ideal for most cyclists, including beginners. The path is within close proximity to emergency services. Ensure you stay on designated cycling lanes for maximum safety.” - Medium Risk (2): “A portion of the route has a medium risk level, suitable for experienced cyclists. While not immediately near emergency services, they are accessible with some delay. Be cautious at intersections and consider wearing high-visibility gear.” Given the low overall risk percentage, the journey is generally safe. However, it's advisable to follow safety tips such as wearing appropriate gear, checking weather conditions, carrying a basic repair kit, staying hydrated, and sharing your route details with someone. Enjoy your cycling adventure in Porto!

Finally, the validation of the method was performed by comparing the responses obtained from the risk map, retrieved from the CityZones tool, to ascertain their consistency. This was an empirical evaluation phase, specifically focused on checking that the method can be used in practical scenarios. This phase presented initial results that were considered as satisfactory. Since we provide a complete implementation in GitHub that is openly accessible for anyone, we believe that additional evaluation results may be also provided by the community in a distributed way, reinforcing the value of the method. Therefore, upon completion of the conducted experiments, we could conclude that the proposed method is a viable approach, with the potential to facilitate the development of promising tools in the context of sustainable and resilient urban mobility. Our method also follows an important recent trend in AI-based tools, reinforcing its timeliness [13,14].

Additionally, our methodology, initially developed and tested in Porto, can be extended to other urban areas, provided certain key conditions are met. These include the availability of comprehensive open geospatial data from the OpenStreetMap, which already has information about cycling infrastructure and points of interest. Additionally, urban risk assessment data provided by the CityZones tool must be accessible, but this tool can already evaluate any city that is present in OpenStreetMap. Furthermore, the target city should have an established or developing cycling infrastructure, including bike lanes and bike-sharing stations, which reinforces the relevance of our method. Since these data are retrieved from open datasets, our methodology and developed tool are easily replicable for any other city.

## Limitations

Although the proposed solution presents a novel approach that uses LLM and open geospatial data to facilitate the analysis of safer cycling routes in urban environments, the novelty of this method poses a set of limitations associated with the use of AI to solve practical problems. These limitations are briefly highlighted as follows.

*GPT-associated limitations:* One significant limitation of GPT-based systems is the potential for artificial hallucinations, whereby the model generates incorrect or misleading responses [15]. For instance, the model may incorrectly interpret the risk distribution along a path and classify a route that is of high risk as a lower-risk route. Nevertheless, by meticulously defining the model instructions (see the context.md file) and adjusting agent Langchain parameters, it is possible to mitigate these hallucinations to some extent.

*Over-reliance on AI:* The proposed method of developing AI systems may result in users becoming overly reliant on them, potentially undermining human judgment and expertise. Therefore, it is crucial to encourage a balanced approach that combines AI assistance with human oversight.

To address the limitations of using GPT-based systems and AI for analyzing safer cycling routes in urban environments, several measures can be implemented. First, to mitigate the issue of artificial hallucinations, it is essential to meticulously define model instructions and adjust parameters, as documented in the context.md file. This precision helps in reducing the likelihood of incorrect or misleading responses, such as misclassifying high-risk routes. Additionally, to prevent over-reliance on AI, a balanced approach that integrates AI assistance with human oversight should be promoted. Enhancing the model's confidence and diversity through comprehensive information inputs will improve system reliability and maintain the crucial role of human judgment and expertise. This combination might ensure that this method is effectively used as a supportive tool rather than replacements for human decision-making.

## Ethics statements

None.

## CRediT author statement

**Daniel G. Costa**: Conceptualization, Methodology, Validation, Formal analysis, Investigation, Writing – original draft. **Ivanovitch Silva**: Conceptualization, Methodology, Validation, Formal analysis, Investigation, Writing – original draft. **Morsinaldo Medeiros**: Conceptualization, Methodology, Validation, Formal analysis, Investigation, Writing – original draft. **João Carlos N. Bittencourt**: Conceptualization, Methodology, Validation, Formal analysis, Investigation, Writing – original draft. **Matheus Andrade**: Methodology, Validation, Formal analysis, Investigation, Writing – original draft.

## Declaration of competing interest

The authors declare that they have no known competing financial interests or personal relationships that could have appeared to influence the work reported in this paper.

## Data Availability

Data will be made available on request.

## Appendix Supplementary materials

Image, application 1
