# Supplementary material for: A method to promote safe cycling powered by large language models and AI agents
Source: MethodsX. 2024 Jul 26;13:102880. doi: 10.1016/j.mex.2024.102880 (PMC11342746; doi:10.1016/j.mex.2024.102880)
Supplement: Supplementary file 1 [file mmc1.pdf]

SoftwareX 23 (2023) 101409

Contents lists available at ScienceDirect

SoftwareX

journal homepage: www.elsevier.com/locate/softx

Original software publication

CityZones: A geospatial multi-tier software tool to compute urban risk zones

João Paulo Just Peixoto [a,b,*], Daniel G. Costa [c,**], Washington de J.S. da Franca Rocha [b], Paulo Portugal [d], Francisco Vasques [c]

[a] IFBA, Federal Institute of Education, Science and Technology of Bahia, Valença, Brazil
[b] PPGM-UEFS, State University of Feira de Santana, Feira de Santana, Brazil
[c] INEGI, Faculty of Engineering, University of Porto, Porto, Portugal
[d] INESC-TEC, Faculty of Engineering, University of Porto, Porto, Portugal

A R T I C L E I N F O

Article history:
Received 20 February 2023
Received in revised form 8 May 2023
Accepted 10 May 2023

Keywords:
Geospatial data
Urban emergency
Smart cities
OpenStreetMap

A B S T R A C T

CityZones is an online tool to support when computing risk zones in a city, which is performed by mathematically evaluating the emergency response capability of an urban area using real data. The tool allows users to define polygonal Areas of Interest (AoI) on a map, which are categorised into low, medium, and high-risk levels. As urban emergencies in general will typically demand the assistance of hospitals (to attend victims), firefighters (for rescue operations and damages reduction), policemen (to control and manage critical situations), and even metro stations (for evacuation), the risk zones within an AoI will be computed according to the distances to such existing infrastructure, which is extracted from the OpenStreetMap database. The coordinates of an AoI are defined at the client-side and submitted to a server as a regular or irregular polygon, which is then stored in a GeoJSON file along with the computed classifications for further processing according to a multi-tier pipeline. Adopting a configurable square-based mathematical model to compute distances and relative risk levels, the risk zones are computed on-demand and exported to CSV files to be used by any application, with an additional representation for web-based visualisation. Moreover, as a practical exploitation of the computed maps, the CityZones tool also suggests ideal positions for sensors-based Emergency Detection Units (EDUs) within an AoI according to the chosen positioning algorithm, assuming that the computed risk zones indicate how severe an undetected emergency would be. While the CityZones tool is not intended to provide a mitigation approach at all, since it does not have knowledge about current emergency situations, it stands as a valuable pre-mitigation tool to be used in the planning phase of emergency management in smart cities, bringing an important contribution for practical applications in this area.
© 2023 The Author(s). Published by Elsevier B.V. This is an open access article under the CC BY license (http://creativecommons.org/licenses/by/4.0/).

Code metadata

Current code version	v1.0
Permanent link to code/repository used for this code version	https://github.com/ElsevierSoftwareX/SOFTX-D-23-00120
Permanent link to Reproducible Capsule	https://cityzones.just.pro.br
Legal Code License	GPLv3
Code versioning system used	git
Software code languages, tools, and services used	Python 3 and JavaScript
Compilation requirements, operating environments & dependencies	POSIX system running a web server with WSGI Phusion Passenger support
If available Link to developer documentation/manual	https://github.com/jpjust/cityzones-v1.0
Support email for questions	joao.just@ifba.edu.br

* Corresponding author at: IFBA, Federal Institute of Education, Science and Technology of Bahia, Valença, Brazil.
** Corresponding author.
E-mail addresses: joao.just@ifba.edu.br (João Paulo Just Peixoto), danielgcosta@fe.up.pt (Daniel G. Costa), wrocha@uefs.br (Washington de J.S. d. Franca Rocha), pportugal@fe.up.pt (Paulo Portugal), vasques@fe.up.pt (Francisco Vasques).

https://doi.org/10.1016/j.softx.2023.101409
2352-7110/© 2023 The Author(s). Published by Elsevier B.V. This is an open access article under the CC BY license (http://creativecommons.org/licenses/by/4.0/).

1. Motivation and significance

The problem of emergency management in cities can be strongly supported by risk maps that identify zones where eventual critical situations are potentially more severe [1]. In practical means, risk maps may indicate the level of urgency for emergency detection in an area, as well as which mitigation services are more required to respond an emergency [2]. With such information, stakeholders could have a more practical perspective of the current distribution of emergency-response facilities in a urban area, as well as planning of emergency management systems could be significantly facilitated. In this scenario, the challenge has been to identify programmable ways to compute risk zones for any defined area in the world, in a cost-effective way, better preparing a city to the occurrence of emergencies. In fact, although the proper mitigation of an ongoing emergency is not in the scope of the tool, the provided information can support other tools with knowledge about emergency mitigation infrastructure.

Given the unpredictable nature of emergency situations, it is important to assess the expected level of resilience of urban areas in the aftermath of an emergency, with a focus on the presence of emergency mitigation facilities. In our previous work [3], we proposed a mathematical model for computing urban risk zones by partitioning an Area of Interest (AoI) into small, continuous squares arranged in a grid-like configuration, which are referred to as Mitigation Zones (MZ). Each MZ is then classified into one of three risk levels (low, medium, or high), based on the relative impact of potential emergencies such as fire, flooding, and earthquakes, and considering the distances from existing mitigation facilities such as fire departments, hospitals, and police stations, each defined by a GPS coordinate as a Point of Interest (PoI). Although our previous work established a flexible mathematical model, further improvements are required to create a more practical tool for addressing real-world problems.

CityZones is an open-source multi-tier platform that allows users to select any regular or irregular polygonal AoI through a web interface, as well as a set of configuration parameters, allowing the computation of risk zone maps based on the existence of different types of mitigation infrastructure. Such selection in Tier 1 is supported by a JavaScript library that directly access data from the OpenStreetMap for visualisation. Users select any configuration parameters and submit a "risk zones computation request" (task) to a cloud-based server through a web interface, which comprises the Tier 2. Then, the received clients' tasks are inserted into a processing FIFO pipeline, with the oldest task being served by the defined algorithms in a sequential way. At a dedicated server at the University of Porto (Tier 3), a file sizing 67 GB containing the metadata of all elements in the OpenStreetMap database was already processed to include only the Points of Interests (hospitals, fire departments, police stations and now also metro stations), for all cities in the world, resulting in a final file sizing 22 GB. This processed file and the clients' requests are the input parameters for the algorithms that will compute the risk zones. The Tier 3 server periodically requests a new task to the cloud-based web server (Tier 2), receiving as response the set of configurations of the first request on the FIFO queue. After running the defined computation algorithms, the Tier 3 server sends back to the cloud server the results in the CSV format containing the classification of each zone, as well as other eventual requested results.

In addition to computing risk zone maps, the CityZones tool can also suggest potential positions for sensor-based Emergency Detection Units (EDUs). To detect emergencies, many cities rely on affordable multi-sensor detection units that monitor various variables and identify abnormal conditions [4,5]. However, deploying a certain number of detection units is not enough; the challenge lies in determining the proportional number of sensors in each region and their optimal position based on the unique characteristics of a city. By classifying different areas according to their risk levels (risk maps), the CityZones tool can compute the proportional risks of each zone and suggest EDU positions accordingly.

This article describes the conceptual structure and the development challenges of the CityZones tool. Practical examples and performance evaluations are also presented, better supporting its adoption in smart city scenarios.

2. Related works

In general, supportive tools to facilitate processing of geospatial data are not a novelty, with some works already presenting valuable results in this area [6–8]. When coming to the processing of city-related metadata, the literature has presented some valuable results of how such tools can be helpful in multiple scenarios [9,10]. Actually, the adoption of crowd-sourced geospatial databases has significantly increased in recent years [11,12], with OpenStreetMap having a great relevance in this scenario as a reliable source of completely access-free geospatial data [13].

Some other works have made proposals to analyse environmental data and classify areas regarding emergencies. In [14], for example, the authors have used the heat-related mortality history in some USA cities to create a correlation between demographic and environmental variables to the risk of dying during hot seasons. The work in [15] proposed a resilience index that makes use of several demographic parameters to classify an area into low, medium or high resilience, creating a resilience map of a city. Another example is the work in [16], that also proposed a resilience index based on demographic and environmental data to classify the resilience of a city.

All these works perform some risk or resilience classification of an area so that it could be leveraged by stakeholders when better planning emergency response actions in a city. However, there are some restrictions: for example, the work in [14] is specific for heat-related mortality, while the works in [15,16] are more generic, but they rely on data from specific regions. In this sense, CityZones comes as a tool that integrates multiple services that are based on previous works, creating a configurable multi-tier processing service that provides risk zones maps for any requesting smart city application, relying on publicly available and unbiased data.

3. Software description

3.1. Software architecture

The defined architecture for the CityZones tool was designed to allow easy access by the users through a powerful but lightweight interface, which is supported by different services with well-defined processing loads. The idea is to achieve a consistent architecture that can be implemented in multiple ways, once the main requirements and conceptual organisation are respected.

The three conceptual tiers of the tool are: Client (Tier 1), Server (Tier 2), and Maps-Service (Tier 3). Each tier will implement one or more software *modules* that will have a well-defined function. Fig. 1 presents such expected flow in 9 processing steps ($S1$ to $S9$).

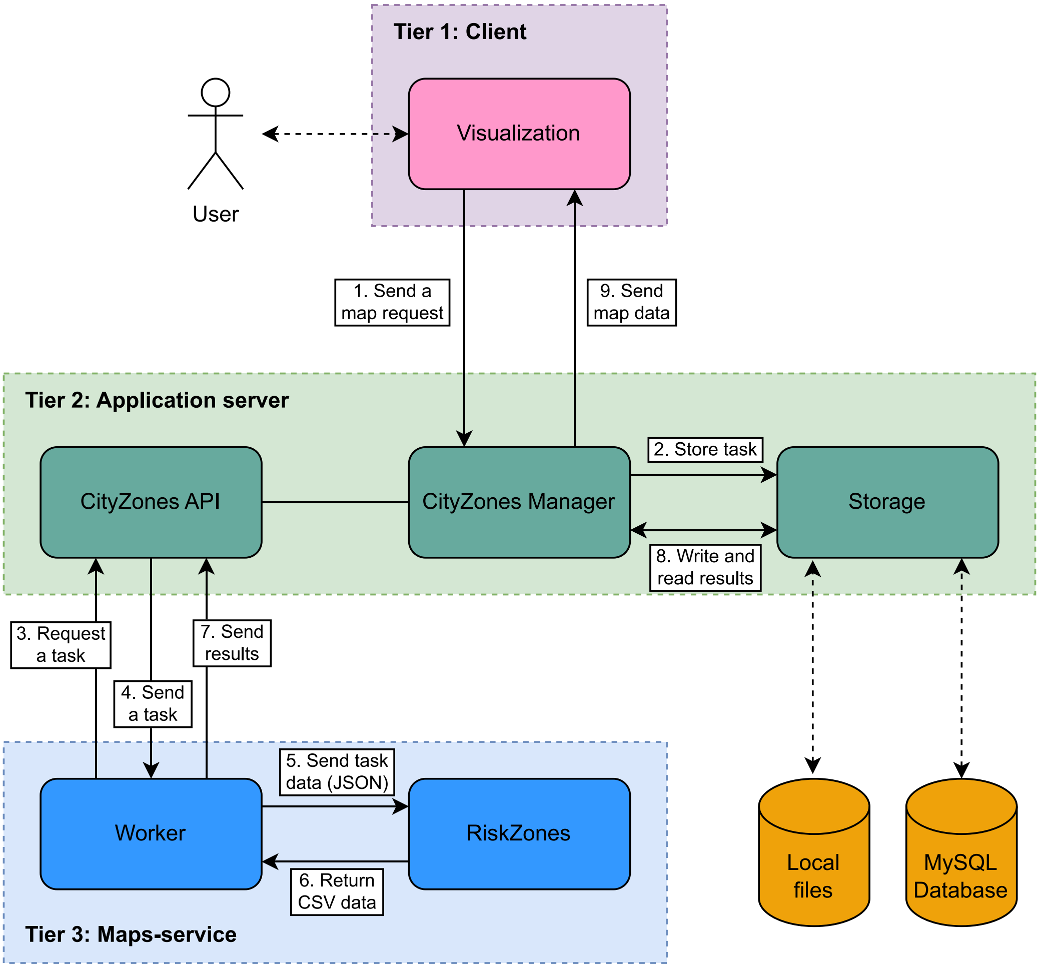

Fig. 1. Processing flow through the defined modules.

3.1.1. Tier 1 - Client

The Client is implemented as a conventional web application to be accessed through a web browser, comprising a visualisation module to handle maps, to display configuration options, and to present the computed results. This module interacts with the application server through the browser, sending requests ($S1$), and receiving computed maps ($S9$) that are then displayed to the user.

The visualisation module will present an interface with a navigable map so that the user can interact with it dynamically, defining the polygon of the AoI and any related parameters (size of the MZ, relevance of each type of PoI, and number of EDUs for deployment). Additional functionalities to facilitate the interaction of the user with the CityZones tool will be also available in this module.

The user interface module was specifically designed to make our proposed algorithms accessible to a wide range of stakeholders, without requiring prior scientific knowledge. This module is recommended for use by public agents involved in city planning, emergency management, and disaster risk reduction, including emergency management coordinators, fire chiefs, police chiefs, EMS (Emergency Medical Services) directors, and directors of public agencies.

3.1.2. Tier 2 - Application server

This tier is implemented as a cloud-based server that is responsible for receiving the users' requests in the form of "tasks", managing a FIFO queue of such received tasks, providing an interface to access them, and transmitting the computed results to the client. A task is a client's request to compute a risk zone, considering all associated metadata for that, which is stored until it is requested by a Maps-service device. This architectural configuration allows the implementation of any number of Maps-services, supporting when multiple clients' requests have to be processed within a short period of time.

A task, which is a core element in the software architecture, can be represented by the tuple (*id*, *created_at*, *requested_at*, *requests*, *description*, *config*, *geojson*), as described in Table 1.

The Application server will be composed of three modules. The first module (CityZones Manager) will handle the overall operation of the server, orchestrating the operation with the other modules. First, it will receive requests to compute risk zones ($S1$) and reply with the corresponding results when they are ready ($S9$). Moreover, the manager module will control the storage of tasks and computed maps, as well as the access to the CityZones API by the Worker module of any number of Maps-service workers. The Storage module will control the access to the database and local storage (steps $S2$ and $S8$), according to the type of data being considered. Finally, the CityZones API module provides the interface so any Maps-service worker receives tasks ($S4$) and sends back the results ($S7$) in an asynchronous way.

3.1.3. Tier 3 - Maps-service

A Maps-service is expected to be implemented on a "powerful" computer to perform the computation of the risk zones, and more than one Maps-service may be implemented to support the CityZones tool.

Table 1
The parameters of a task.

Parameter	Description
id	The ID of the task, used for its identification when sending and receiving tasks from a Maps-service worker
created_at	The timestamp of the creation time of the task
requested_at	The timestamp of the last time it was sent to a Maps-service worker
requests	How many times this task was sent to a Maps-service worker
description	The user-defined description of the task
config	The JSON configuration of the task (contains the boundaries of the grid, zone length, number of EDUs, positioning algorithm and weights of the PoIs)
geojson	The GeoJSON defining the polygon of the AoI

Two modules are implemented by a Maps-service. The first module (Worker) sends a request for a task ($S3$) and receives the definition of a task to be processed ($S4$) from the application server. After $S3$, it starts processing the received task, extracting the AoI from the OpenStreetMap global data. Then, the second module (RiskZones) is executed (5), which computes the risk zones for the AoI and (if requested) the positions of the EDUs for the set of computed zones. Once the results are ready for a particular task ($S6$), the Maps-service sends it back to the application server ($S7$), asynchronously, which will store the resulting CSV files (computed maps) on its local storage ($S8$), as well as any associated metadata to the MySQL database.

3.2. Software implementation

Different technologies and programming tools were employed to implement CityZones. The Client is a web application that users can access through a web browser, requiring a minimum support to JavaScript. The map interface displayed on the web client is powered by Leaflet [17], a JavaScript library that provides a graphical interface for maps. When results are ready, the JavaScript code (retrieved from the application server and executed on the browser) will load the OpenStreetMap image data for map previewing, allowing very intuitive and visual interactions with the users.

The Application server was written in the Python 3 programming language on the Flask framework [18], which provides an easy-to-start environment for web applications. The server codes run on the cloud-based hosting service Dreamhost [19], although any cloud hosting service or UNIX-like server with Python 3 and WSGI Phusion Passenger [20] support for application loading could be employed. Concerning the implemented database, the available MySQL service [21] on the same hosting service was adopted to store the tasks and other relevant metadata. The Application server is a monolithic application that provides the views for the Client.

The Maps-service modules (Worker and RiskZones) are also written in the Python 3 programming language. Concerning this Tier, the Worker module loops forever, while the RiskZones module is executed when requested. The interactions between the Worker module and the Application server through its CityZones API are performed through the REST API.

Any Linux system that supports Python 3, the Osmium command line tool [22] and with the complete OpenStreetMap PBF data file can run the Maps-service modules. To avoid vandalism and unauthorised access, an API key must be set in both the Application server and the Maps-service Workers, so only systems with the correct key can request tasks and transmit results.

3.2.1. Files formats

The configuration for the RiskZones module is given by the Application server in the JSON format (JavaScript Object Notation) [23], while the AoI delimitation is provided by the Application server in the GeoJSON format [24].

When an user requests a new task on the map view, CityZones creates a JSON configuration file and a GeoJSON of the AoI so that the RiskZones tool can use them to process the classification and EDUs positioning. This processing is performed on the Maps-service side. The CityZones tool provides access to the JSON and GeoJSON files generated through the download link that appears at the task's row on "Tasks" view, indicated with a down-arrow on a file icon. By clicking in the configuration download link, CityZones will send to the browser a ZIP file containing both JSON and GeoJSON files so that the user can decompress it and run RiskZones tool via command line (if desired). These files allow the user to make modifications to the configuration of the task and run it locally. It is important to notice that this approach requires an OSM or PBF file containing the AoI to be classified. Such file can be exported via OpenStreetMap web interface or downloaded from Planet OSM [25].

Another download link indicated by a CSV acronym on a file icon allows the user to download the results of the task. The downloaded ZIP file contains three files: the map CSV for the zones classification, the EDUs CSV for the EDUs positions and the roads CSV for the roads and streets detected in the AoI (this data is used for EDUs restricted positioning). These CSV files can be imported as assets into Google Earth Engine or any other GIS software.

3.2.2. Computing risk zones

The first step that a Maps-service worker will take after receiving a task is to get its AoI polygon to extract its data from the OpenStreetMap reference data file. That data file is a 67 GB PBF (Protocolbuffer Binary Format) file containing data of the whole world, directly downloaded from the OpenStreetMap collaborative database. Actually, it can be reduced manually by running *osmfilter*, a OpenStreetMap command line tool that filter data from a PBF or OSM file, potentially facilitating processing of geospatial data based on it. Since the initial version of the CityZones tool is currently considering only hospitals, fire stations, police stations, metro stations and roads for classifying zones and positioning EDUs, the file that is actually used by a Maps-service worker is a filtered PBF file with 22 GB of data.

For AoI extraction, a Maps-service worker uses another command line tool from OpenStreetMap: *osmium* [22]. Its Worker module sends the top, bottom, left and right boundaries of the AoI to the osmium tool, so it can extract this specific area from the original data file and save it into another file for further processing.

The RiskZones module makes use of multiprocessing to accelerate the performance of the classification step, using the available cores of the processor and a proper Python library (MultiProcessing). When that module finishes, the corresponding Maps-service worker sends the data to the Application server via its REST API.

Finally, although some initial definitions for the RiskZones module are described in [3], we considerably extended that to incorporate new elements, also making it modularised for eventual

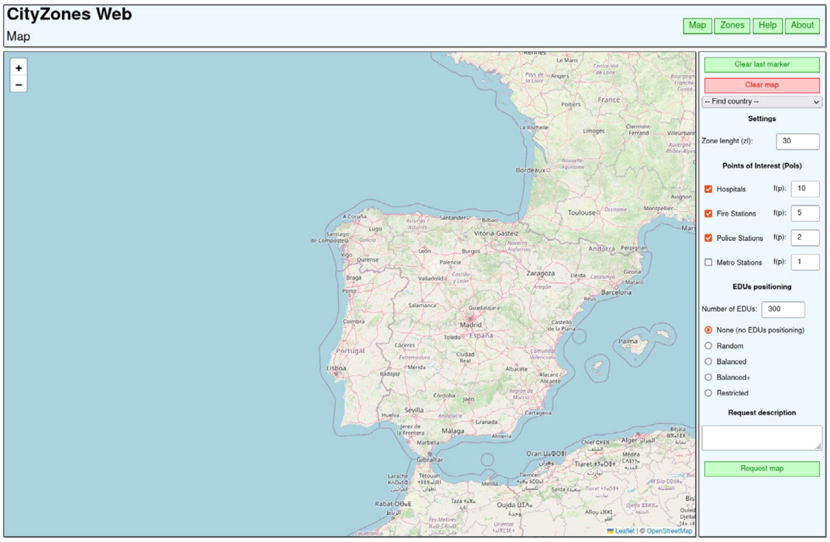

Fig. 2. A screenshot of the initial page of the CityZones web page.

extensions. When starting the intended classifications, a major change was to consider the definition of an AoI that could be any regular or irregular polygon, instead of only a rectangle as in [3]. The first processing step is then to check the zones inside the polygon of the AoI, considering a grid of square zones that is previously defined based on a rectangle circumscribing the AoI polygon. For each zone in the grid, this module checks if it is inside the polygon, flagging the zone object as *inside*. This is a very time-consuming task because for each zone in the grid the algorithm has to check for an intersection between an imaginary line from that zone to the infinite and every line segment that makes the polygon of the AoI. The same is done for each PoI. Although the high computational costs, this procedure assures higher flexibility to the users when defining the areas of interest (polygons are better to accurately define zones than squares).

After computing the list of valid zones and PoIs (the ones inside the AoI), the computation of the risk levels can be performed as described in [3]. Based on that work, two modifications were made to the computation of the risk zones and EDUs positions. The first one was changing the risk perception computation from a logarithm of base 10 to a natural logarithm (base e). In fact, the use of a natural logarithm is more suitable for the problem of classification within classes in a context of urban risks [26, 27], particularly due to its mathematical properties. Secondly, we extended the EDUs positioning algorithms to take streets as permitted deployment areas.

3.3. Software functionalities

The interactive part of the CityZones software tool is the web application interface. The main page of the application shows an interactive map and a toolbar at the right, as shown in Fig. 2. In that figure, the user can mark points on the map by clicking on it, creating the AoI polygon. When the user clicks the first mark again, a polygon is closed and the AoI is defined.

The toolbar allows the user to clear the visualisation map and start it from scratch, to clear the last mark in case of a mistake, or to upload a GeoJSON file for AoI definition. The most important features are in the settings area of the toolbar, which contains the options for the task processing. The user can select the parameters of the classification: the zone length, which PoIs will be considered and their relevance weight ($f(p)$), the number of EDUs to position, and which positioning algorithm the RiskZones module will use (we implemented four different algorithms). At the bottom of the settings bar there is a input area so the user can write a description of the task for debugging and tracking.

Fig. 3 presents the map view with an user-defined AoI for the city of Porto, Portugal.

After defining an AoI and the configuration of a task, the user can submit it by clicking on "Request map" button. The "Tasks view" function in the Client shows all the submitted tasks and their status. New tasks are in the QUEUED state, meaning that the task is ready to be processed, but no Maps-service worker has requested it yet. When a task is assigned and transmitted to a Maps-service, it goes to the PROCESSING state, considering that a single task is assigned to no more than one Maps-service worker.

If a task takes more than one hour to be processed, it is assumed as EXPIRED and returns to the queue of the Application server. This allows the reassignment of a task to any requesting Maps-service worker. Additionally, when the same task is assigned three times in a row, with no computed result, it is assumed as FAILED. This may happen when the configuration is somehow invalid, or when a too large or incorrect AoI polygon is defined.

When a task is finished and the Application server receives the corresponding results, it gets into the DONE state. After that, a user can click the "Show map" button in the tasks list to view the resulting classification and EDUs position on map. If needed, the user can also download both the configuration used by the RiskZones module and the result CSV files generated by it using the two links at the task row. The configuration files can be used locally by the user in the RiskZones module and the CSV files can be used in other GIS softwares, including QGIS [28] and Google Earth Engine [29]. Also, the list provides a third link that allows the user to repeat the same AoI in another task.

The tasks list in the "Tasks view" area displays information about the task. An example of that is presented in Fig. 4.

After clicking the "Show map" button to visualise the results of a classification, a heat-map for the AoI classification is shown, with red being the worst configuration while green is the best. A slider at the bottom of the list allows the user to set the opacity of the heat-map for proper visualisation on the map. Also, a "Download map image" button is provided so the user can download a PNG image file of the map shown on the screen. Finally, when properly indicated by the user, the computed map will present the position of the EDUs on the AoI, which are indicated by black dots.

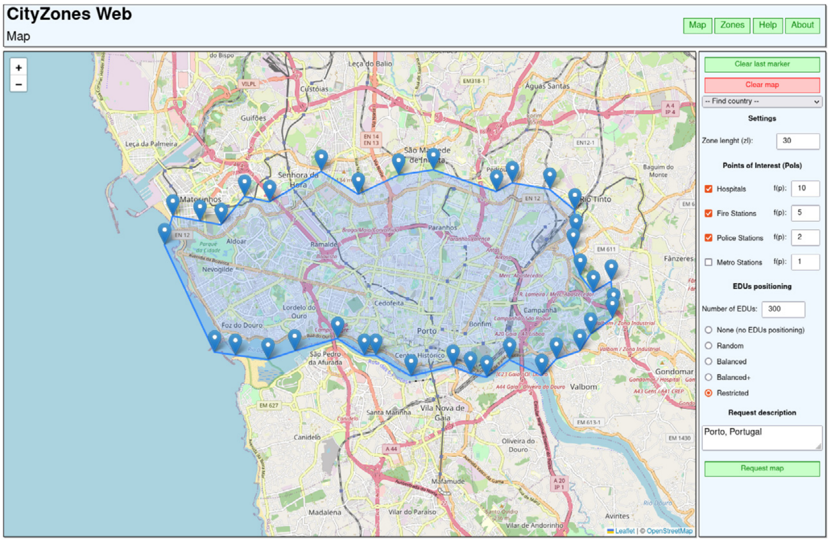

Fig. 3. A screenshot presenting a defined AoI for the city of Porto, Portugal.

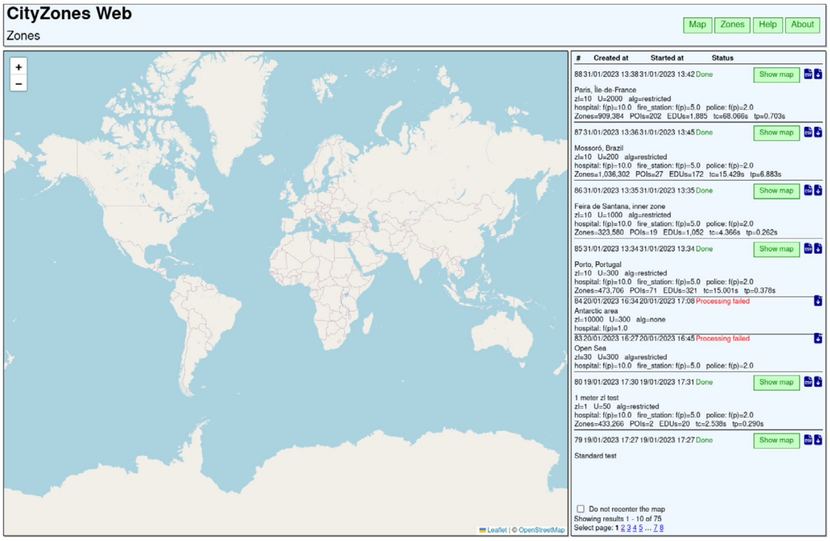

Fig. 4. A screenshot presenting a list of task with different status.

4. Illustrative example

4.1. Risk zones in Berlin

The presented example in this section is based on the city of Berlin, Germany, which has a total area of 891.82 km^2. For that city, any configuration of an AoI is possible, comprising only the area of Berlin or even spreading across multiple adjacent cities. Actually, as an AoI can be any polygon, we can define it by surrounding the Berlin's perimeter, delimiting only this area to define a task, which results in an AoI containing 50 points.

For the initial defined task, the settings in Table 2 were considered.

After defining the settings, the first task was submitted using the proper button on the CityZones web interface. After being stored by the Application server, it was eventually retrieved by a Maps-service worker, which processed it and returned the final results when concluded. Those results was finally delivered to the Client, being presented as shown in Fig. 5(a), for a total of 1,199,092 computed zones and 239 PoIs.

The number of zones in an AoI depends not only on its area, but also on the zone length (*zl*). In this scenario, the zone length

Table 2
Parameters for the initial defined task in these examples.

Parameter	Value	Description
zl	30 m	Size of a squared-sized zone
U	2.000	The total number of EDUs to deploy
$f(hospital)$	10	Relevance weight of hospitals
$f(fire\_station)$	5	Relevance weight of fire departments
$f(police\_station)$	2	Relevance weight of police stations
$f(metro\_station)$	0	Relevance weight of metro stations
Algorithm	*restricted*	EDUs positioned on streets

was 30 m. For more detailed results, smaller zones could be used, but more zones would be generated for the same area (higher computational cost). For instance, another test was performed with a zone length of 10 m for the same AoI, which resulted in an AoI with 10,797,265 zones. It is possible to set the zone length as lower as 1 meter, but the amount of memory it would need might terminate the RiskZones process due to hardware limits of the running device.

A second test was also conducted for the same AoI. In this test, some parameters were changed: the number of EDUs (U

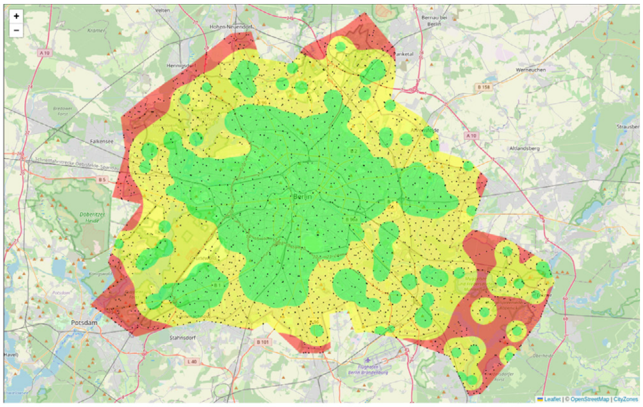

(a) Results for the initial CityZones classification.

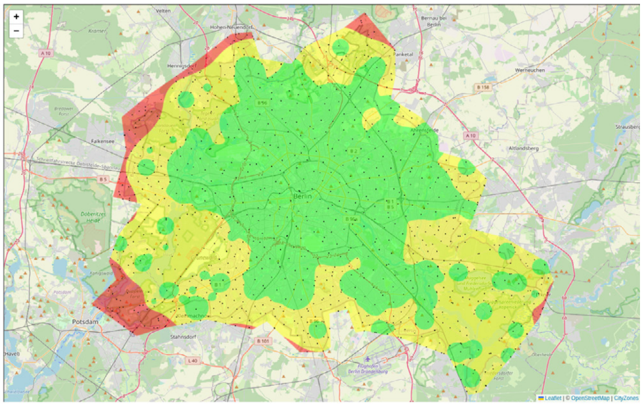

(b) Results for the second task.

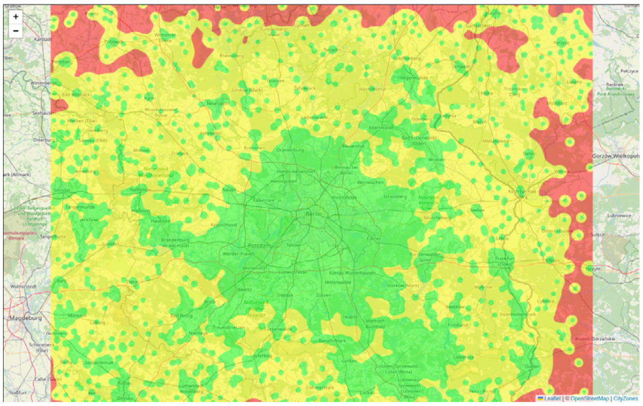

(c) Results for the third task.

Fig. 5. Computed classifications for Berlin. Red areas represent high risk, yellow areas represent medium risk, and green areas represent low risk. (For interpretation of the references to colour in this figure legend, the reader is referred to the web version of this article.)

parameter) was reduced by half, while the weight of the PoIs was changed in order to intentionally have a PoI type with a much higher weight than the others, in this case, the fire departments, with results depicted in Fig. 5(b).

It is noticeable that the heat-map has changed, prioritising the fire station PoIs, thus, zones that were in red in Fig. 5(a) get in yellow or green in Fig. 5(b) if they are near fire stations. This example demonstrates how different results may be produced depending on the settings of the task.

As a third example of using the CityZones tool, a very large AoI was defined surrounding the city of Berlin and taking parts of its metropolitan area, comprising a total area of 35,987.16 km^2 and with zones sizing 100 m. Moreover, we set $f(hospital) = 10$, $f(fire\_station) = 5$, $f(police\_station) = 2$, and $f(metro\_station) = 0$, with $U = 0$ (no EDUs positioning was requested). The results for this configuration are presented in Fig. 5(c), totalling 3,598,716 processed zones for 1,884 PoIs.

The distribution of zones are now different from the other examples because of the change in the AoI. Since the risk levels assignment are relative to the whole area, it is expected that different classifications are obtained with different areas.

4.2. Performance evaluation

The CityZones tool can be implemented in any hardware that supports minimum communication and processing functions, but it is recommended that reasonable processing power is available specially when multiple tasks are requested within a period of time. Currently, the Application server is hosted in a regular cloud-based service as it does not demand much processing power. However, since a Maps-service device will have to process the zones and PoIs for each AoI, also computing the risk zones, it should have reasonable memory and processing resources. An initial Maps-service device was implemented in a computer with an Intel i9 12th generation processor, 64 GB DDR5 RAM memory, and 2TB of SSD storage, which is located at the University of Porto. Actually, while the Application server is publicly accessible on the Internet, a Maps-service can operate under any NAT-based network because tasks are requested by it to the Application server, asynchronously. Moreover, as a multi-core processor was selected to be used by the reference Maps-service device, the MultiProcessing Python library is used by the RiskZones module to achieve parallel processing and reduce the overall computation time with the cost of using 100 % of all CPUs. Regarding the memory usage, the RiskZones module create a list of zones to manage the data of the AoI with each zone consuming 272 bytes, thus, the amount of memory usage depends on how many zones will be instantiated by the module, which is directly related to the area of the AoI and the length of each zone.

Since all the processing is performed in the Maps-service device, the required time for risk classification and EDUs positioning depends on the computer system that is processing the task. Table 3 shows some results for tasks processed on the reference Maps-service.

Although the final processing time depends on the available hardware resources, the performance of the RiskZones module also depends on the number of zones, PoIs and points that form the AoI polygon. Moreover, regarding the performance of EDUs positioning using the restricted positioning algorithm [3], the arrangement of the allowed zones (in this case, the position and form of streets in the AoI), will also influence the time it takes to finish the processing.

It is worth noting that Tiers 1 and 2 do not perform any processing for the classification and positioning steps. Tier 1 only serves as an interface for sending and viewing tasks, while Tier 2 acts as a central data repository, storing and providing task data. Therefore, all performance metrics are measured in Tier 3, where the actual processing takes place.

5. Impact

The CityZones tool may have a significant impact on emergency management in cities. In an initial perspective, it provides stakeholders with an essential tool to identify areas that require urgent attention in case of an emergency. With the risk maps

Table 3
Performance of executed tasks, with t_c as the classification time and t_p as the EDUs positioning time.

Region	AoI points	Zones	PoIs	EDUs	t_c (s)	t_p (s)
Berlin, DE	50	1,199,092	239	2,014	103.516	275.158
Berlin surroundings	4	3,598,716	1,884	0	2,324.803	–
Paris, FR	26	909,383	161	2,024	53.915	144.984
Porto, PT	39	473,705	60	305	12.472	24.340
Feira de Santana, BR	18	355,556	19	1,002	4.059	34.061

produced by the tool, decision-makers can easily determine the priority of emergency detection in different regions, and plan for mitigation services accordingly. This information is crucial in improving the preparedness of cities for emergencies, which is a critical aspect of public safety. In fact, by identifying the potential risks of different areas in a city, emergency responders can better prepare for potential emergencies and allocate their resources more effectively, potentially leading to faster response times and more efficient use of resources, ultimately saving lives and minimising damage.

The CityZones tool not only helps in understanding the current resilience of cities to emergencies but also extends its impact by identifying potential positions for sensor-based Emergency Detection Units (EDUs). With the efficient deployment of these units, the tool can further improve emergency management systems that operate around the active detection of critical situations by real-time sensing.

Overall, the CityZones tool has great potential for improving the resilience of urban areas in emergency situations, making it a valuable asset for smart city scenarios. The scalability of the tool, enabled by cloud-based servers for processing risk zone computations, makes it capable of handling multiple requests at once. Additionally, the use of data from the constantly updated OpenStreetMap database means that the tool can be used for cities worldwide. Given these advantages, we consider CityZones to be a significant contribution to the field.

6. Conclusion

The computation of risk maps has wide-ranging implications for various applications, including emergency rescuing, urban planning, smart mobility, real estate valuation, public security reinforcement, crisis management, and disaster avoidance. Therefore, having a reliable computational tool to support the computation of such maps can have a significant impact on how these applications are used in practical scenarios, benefiting society as a whole.

The CityZones tool was implemented as a multi-tier modularised tool with a well-defined programming interface between the modules. This design enables the inclusion of new modules to support additional services. For instance, the AoI definitions and general maps handling can be completely reused by new services, which is one of the design requirements of the tool.

As future works, the CityZones tool will be extended to incorporate new EDUs positioning algorithms based on research works in the literature. Additionally, other types of PoIs will be also considered, taking for example major road junctions and real-time positions of response vehicles as new mitigation-related input parameters.

Declaration of competing interest

The authors declare that they have no known competing financial interests or personal relationships that could have appeared to influence the work reported in this paper.

Data availability

Data will be made available on request

Acknowledgment

This work was supported by national funds through the FCT/MCTES (PIDDAC), under the project EXPL/EEI-COM/1089/2021. It is also supported by INEGI-LAETA (FCT project UIDB/50022/2020). Finally, this work was financed in part by the Coordenação de Aperfeiçoamento de Pessoal de Nível Superior - Brasil (CAPES) - Finance Code 001.

Appendix A. Supplementary data

Supplementary material related to this article can be found online at https://doi.org/10.1016/j.softx.2023.101409.

References

[1] Cariolet J-M, Vuillet M, Diab Y. Mapping urban resilience to disasters – A review. Sustainable Cities Soc 2019;51:101746.
[2] Costa DG, Peixoto JPJ, Jesus TC, Portugal P, Vasques F, Rangel E, et al. A survey of emergencies management systems in smart cities. IEEE Access 2022;10:61843–72.
[3] Peixoto JPJ, Costa DG, de J. S. da Franca Rocha W, Portugal P, Vasques F. Optimizing the deployment of multi-sensors emergencies detection units based on the presence of response centers in smart cities. In: 2022 IEEE international smart cities conference. Pafos, Cyprus; 2022, p. 1–7.
[4] Oliveira F, Costa DG, Silva I. On the development of flexible mobile multi-sensor units based on open-source hardware platforms and a reference framework. HardwareX 2021;10:e00243.
[5] Avgeris M, Spatharakis D, Dechouniotis D, Kalatzis N, Roussaki I, Papavassiliou S. Where there is fire there is SMOKE: A scalable edge computing framework for early fire detection. Sensors 2019;19(3).
[6] Almeida de Macedo Oliveira AA, Hirata R. INACITY - Investigate and analyze a city. SoftwareX 2021;15:100777.
[7] Solís P, Anderson J, Rajagopalan S. Open geospatial tools for humanitarian data creation, analysis, and learning through the global lens of YouthMappers. J Geogr Syst 2021;23(4):599–625.
[8] Bernardini G, Quagliarini E, D'Orazio M, Brocchini M. Towards the simulation of flood evacuation in urban scenarios: Experiments to estimate human motion speed in floodwaters. Saf Sci 2020;123:104563.
[9] Cai J, Huang B, Song Y. Using multi-source geospatial big data to identify the structure of polycentric cities. Remote Sens Environ 2017;202:210–21.
[10] Borgogno-Mondino E, Lessio A. Geospatial tools in support of urban planning: A possible role of historical maps in programming a sustainable future for cities. In: International conference on computational science and its applications. Cagliari, Italy: Springer; 2020, p. 649–63.
[11] Basiri A, Haklay M, Foody G, Mooney P. Crowdsourced geospatial data quality: Challenges and future directions. Int J Geogr Inf Sci 2019;33(8):1588–93.
[12] Coetzee S, Ivánová I, Mitasova H, Brovelli MA. Open geospatial software and data: A review of the current state and a perspective into the future. ISPRS Int J Geo-Inf 2020;9(2).
[13] Minghini M, Frassinelli F. OpenStreetMap history for intrinsic quality assessment: Is OSM up-to-date? Open Geospatial Data, Softw Standards 2019;4(1):1–17.
[14] Hondula DM, Davis RE, Saha MV, Wegner CR, Veazey LM. Geographic dimensions of heat-related mortality in seven U.S. cities. Environ Res 2015;138:439–52.
[15] Hung H-C, Yang C-Y, Chien C-Y, Liu Y-C. Building resilience: Mainstreaming community participation into integrated assessment of resilience to climatic hazards in metropolitan land use management. Land Use Policy 2016;50:48–58.
[16] Kontokosta CE, Malik A. The resilience to emergencies and disasters index: Applying big data to benchmark and validate neighborhood resilience capacity. Sustainable Cities Soc 2018;36:272–85.
[17] Crickard III P. Leaflet.Js essentials. Packt Publishing Ltd; 2014.
[18] Aggarwal S. Flask framework cookbook. Packt Publishing Ltd; 2014.

[19] Dreamhost web hosting. 2023, https://www.dreamhost.com. [Online; Accessed 07 February 2023].
[20] Passenger - enterprise grade web app server for ruby, Node.js, Python. 2023, https://www.phusionpassenger.com/. [Online; Accessed 07 February 2023].
[21] MySQL database. 2023, https://www.mysql.com. [Online; Accessed 07 February 2023].
[22] Osmium tool. 2023, https://wiki.openstreetmap.org/wiki/Osmium. [Online; Accessed 07 February 2023].
[23] Introducing JSON. 2023, https://www.json.org. [Online; Accessed 07 February 2023].
[24] GeoJSON. 2023, https://www.geojson.org. [Online; Accessed 07 February 2023].
[25] Planet OSM. 2023, https://planet.openstreetmap.org/. [Online; Accessed 07 February 2023].
[26] Williams MD, Jean MC, Chen B, Molinari N-AM, LeBlanc TT. Primary care emergency preparedness network, New York City, 2015: Comparison of member and nonmember sites. Am J Public Health 2017;107(S2):S193–8.
[27] Cardona OD, Ordaz MG, Yamin LE, Marulanda MC, Barbat AH. Earthquake loss assessment for integrated disaster risk management. J Earthq Eng 2008;12(S2):48–59.
[28] QGIS: A free and open source geographic information system. 2023, https://qgis.org/en/site/. [Online; Accessed 07 February 2023].
[29] Google earth engine. 2023, https://earthengine.google.com. [Online; Accessed 07 February 2023].
